# Ambiguity preferences in intertemporal and risky choice: A large-scale study using drift-diffusion modelling

**DOI:** 10.3758/s13423-025-02709-2

**Published:** 2025-06-25

**Authors:** Mingqian Guo, Iris Ikink, Karin Roelofs, Bernd Figner

**Affiliations:** 1 Behavioural Science Institute, Radboud University, Thomas Van Aquinostraat 4, 6525GD Nijmegen, The Netherlands; 2 Donders Institute for Brain, Cognition and Behaviour, Radboud University, Nijmegen, The Netherlands; 3 Department of Experimental Psychology, Ghent University, Ghent, Belgium

**Keywords:** Ambiguity, Intertemporal choice, Risky choice, Drift-diffusion model

## Abstract

Intertemporal choices constitute a significant topic of interest in both psychological and behavioral-economics research. While many studies focus on decisions with precisely known reward delivery times, real-world situations typically involve only an imprecise knowledge of these timings (i.e., the delivery times are ambiguous). The current study uses a large size dataset (sample size *N* > 669) consisting of both risky and intertemporal ambiguous and nonambiguous choices and aims (i) to clarify the relationship between probability-ambiguity and time-ambiguity effects on choice, and (ii) to evaluate different computational models (attribute-wise and integrated-value models) across risky and intertemporal choice domains using a drift-diffusion model (DDM) framework. Analysis of the choice data revealed a significant association: Individuals who were more averse to time ambiguity also exhibited a stronger aversion to probability ambiguity, as indicated by a correlation of *r* = .28. The DDM analyses revealed that (i) DDMs incorporating ambiguity preferences outperformed models without ambiguity preferences in both the time and probability domain for most participants. Interestingly, (ii) while time-ambiguity aversion was best explained by an attribute-wise model, probability-ambiguity aversion was best explained by an integrated-value model. Finally, we found that (iii) if an individual’s intertemporal decisions were best explained by a DDM incorporating ambiguity, then their risky decisions were also most likely best explained by a DDM incorporating ambiguity.Taken together, our results are evidence that ambiguity preferences across the time and probability domains are not independent but show some consistency despite the differing—attribute-wise versus integrated-value—decision strategies in each domain.

**Supplementary Information:**

The online version contains supplementary material available at 10.3758/s13423-025-02709-2.

## Introduction

In the decision-making literature, *ambiguity* refers to situations where information is unknown or imprecisely defined (Ellsberg, 1961; Golman et al., 2021). Such ambiguity is commonly encountered in daily life. For example, choosing a new restaurant with no online reviews is an example of a risky decision under ambiguity, as one does not know the probability of being served good food. The most widely studied form of ambiguity in decision-making is what we call probability ambiguity (i.e., decisions in which the probability of one or several outcomes are partially or completely unknown). Such probability ambiguity has been extensively investigated in the context of risky choice, and research suggests that individuals typically exhibit probability-ambiguity aversion (i.e., reduced preferences for options with ambiguous probabilities). Developmental studies have indicated that adolescents’ greater propensity for risk-taking may stem from higher ambiguity tolerance (Tymula et al., 2012). Moreover, findings from psychiatric research suggest that individuals with stronger probability-ambiguity aversion may be less likely to engage in behaviors with uncertain outcomes, such as substance use, smoking, or heavy alcohol consumption (Dalley et al., 2011; Saposnik et al., 2016).

Beyond risky decision-making, ambiguity also plays a role in decisions related to time. In everyday life, we frequently choose between options that yield rewards or outcomes at different points in the future. Such temporal decisions are typically studied in the laboratory using intertemporal choice tasks (e.g., participant might be asked whether they prefer €5 now or €10 in 100 days). However, these tasks typically assume precise knowledge of waiting times (Frederick et al., 2002). In reality, we are often uncertain about exactly when future events will occur—a situation we will refer to as *time ambiguity*. Traditional intertemporal choice tasks typically neglect this critical feature of real-world decision-making.

Accordingly, time ambiguity has only recently begun to receive attention. Ikink et al. (2019, 2023) introduced time ambiguity into intertemporal choice tasks and found that, on average, participants are averse to time ambiguity, preferring options with known delays over those with ambiguous waiting periods. The current study has two main focuses: first, exploring the connection between these two types of ambiguity preferences, and second, comparing a series of computational models across both domains.

Ambiguity preferences in both risky and delay discounting may be driven by shared cognitive and affective processes. For instance, tolerance for uncertainty is a broad trait that can manifest across different domains of decision-making. Individuals with a lower tolerance for uncertainty may avoid both ambiguous probabilities and ambiguous delays, leading to correlated ambiguity preferences. And neuroimaging studies show that time ambiguity elicits activation patterns similar to those observed in probability ambiguity, engaging cognitive control regions such as the intraparietal sulcus (Ikink et al., 2019; Krain et al., 2006; Levy et al., 2010). However, it remains unclear whether an individual’s time-ambiguity preferences are related to their probability-ambiguity preferences. Establishing such a connection is important both theoretically—potentially revealing domain-independent ambiguity attitudes since these two ambiguity preferences not only have similar concept but also similar neural pattern—and practically, as it may help predict real-world behaviors across different domains.

Computational modeling can advance our understanding of the cognitive processes underlying these decisions. Integrated-value models, such as hyperbolic discounting or prospect theory assume that individuals integrate relevant attributes (e.g., amount, delay, probability) into a single subjective value (Perkins & Rich, 2021). These models have historically dominated research on both risky and intertemporal decision-making. However, emerging evidence suggests that attribute-wise models, which assume that individuals compare attributes separately rather than combining them into a single value, may better capture the complexity of human decision-making (Ericson et al., 2015; Glickman et al., 2019). Ikink et al. (2019, 2023) relied solely on integrated-value models to explain time-ambiguity preferences. Similarly, in studies of probability ambiguity, the most commonly used Gilboa–Schmeidler model also falls under the category of integrated-value models (Gilboa & Schmeidler, 1989). This raises the question of whether attribute-wise models could provide a better account of ambiguity preferences. Compared with integrated-value models, attribute-wise models naturally account for various anomalies observed in intertemporal choice tasks, such as magnitude and super-/subadditivity effects (Scholten & Read, 2010). Furthermore, research on individual differences indicates that individuals with greater patience are more likely to rely on attribute-wise models when making intertemporal decisions (Amasino et al., 2019; Reeck et al., 2017).

In the current paper, we analyzed of a substantial data set that investigated time ambiguity and probability ambiguity in a within-subjects design. We employed drift-diffusion modeling (DDM) in combination with generalized linear-mixed effects modeling (GLMM) to examine the association between ambiguity preferences in the probability and the time domain (delay domain). DDM is a type of sequential sampling model (SSM) that conceptualizes decision-making as a process of noisy evidence accumulation over time, and it has been widely applied to both response time (RT) and choice data (Ratcliff et al., 2016).

To summarize, we combined GLMM and DDM approaches to analyze a large sample data set to offer a more comprehensive view of the link between ambiguity preferences in the probability and time domains. First, we investigate the choice data using GLMM, which allows us to assess the impact of variations in attributes such as ambiguity levels, delays, and probabilities on choice, as well as to assess the potential associations between probability-ambiguity preferences and time-ambiguity preferences. As a critical next step, we compare a series of attribute-wise models as well as integrated-value models using DDM. To examine individual differences in and associations between ambiguity preferences across the probability and time domains, we use model selection metrics, such as Bayesian model averaging (BMA).

## Methods

### Participants

Data (choices and RTs) were collected online using Qualtrics software; participants were recruited at Radboud University via Sona. In total, 1,128 students (mean age: 20.6 years; 76% women) participated in the experiment in exchange for course credit. A subset of the data was used to assess the suitability for participating in an fMRI experiment (Ikink et al., 2025). Prior to participating in the study, participants gave informed consent. The substantial sample size in our study ensures that we should have sufficient statistical power to examine the association between the two types of ambiguity preferences. The study was pre-registered on the Open Science Framework (OSF). The preregistration details, including the study design, hypotheses, and planned analyses, can be accessed online (https://osf.io/bwnxh/).

We hypothesize that the two types of ambiguity preference are connected. In the preregistration, we initially planned to analyze individual differences solely with a mixed-effects model. However, we have extended the analysis to include computational modeling, as this approach incorporates both RT and choice data, potentially offering a more accurate representation of participants’ preferences.

### Experimental design

Each trial presented two choice options: a fixed, hidden option of €5 available immediately, and a variable option displayed centrally whose reward magnitude and delay/probability were either exact or ambiguous. Delay blocks contained only intertemporal choice trials, while probability blocks included only risky choice trials. Participants completed 210 trials in total, organized into 14 blocks of 15 trials each, alternating between delay and probability blocks.

**Fig. 1:**
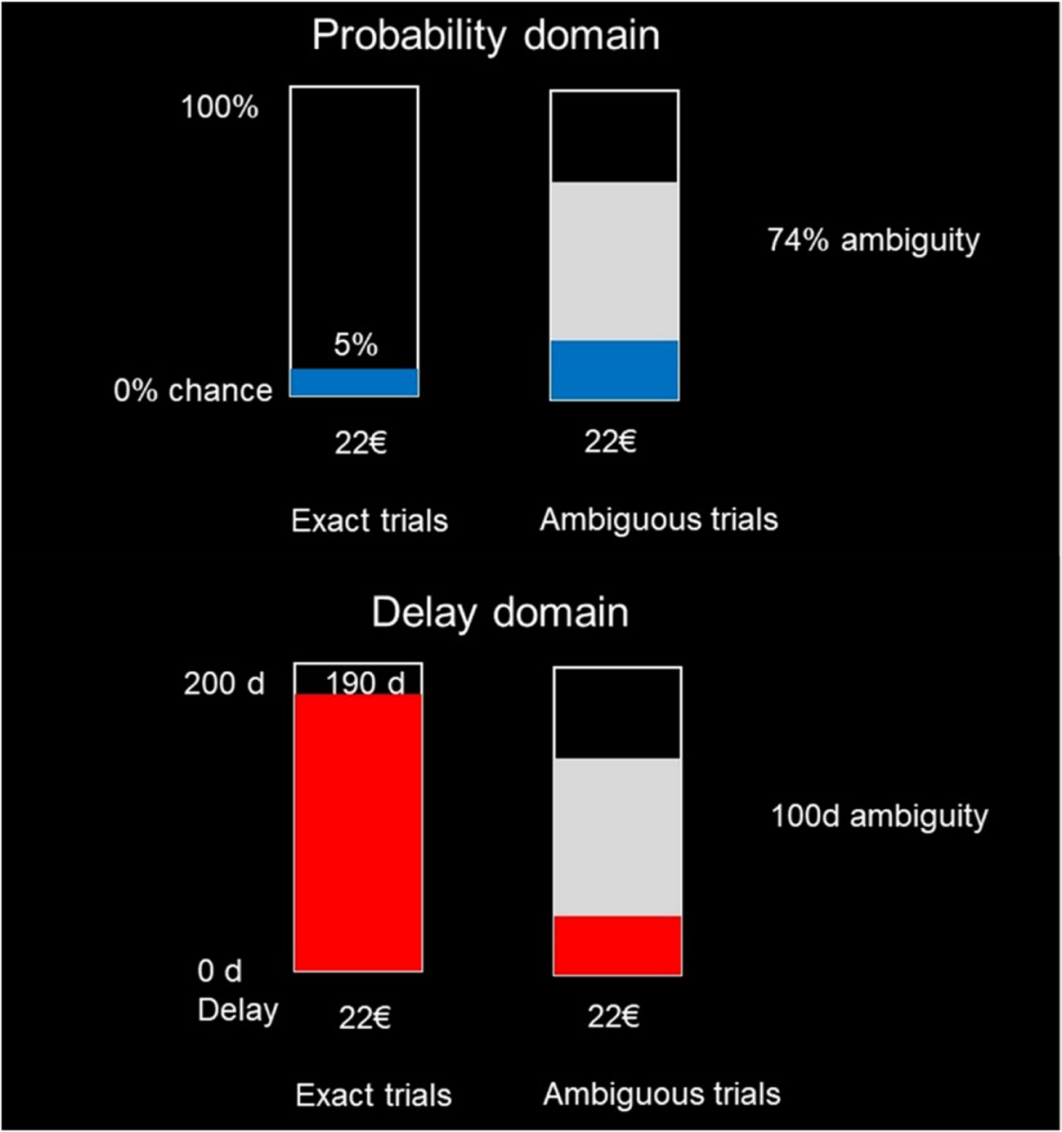
Example of risky and intertemporal choice trials. *Note.* Participants engaged in two types of decision tasks in the experiment, a risky and an intertemporal choice task. In both tasks, they were asked to choose between the option shown on the screen (which varied across trials and was either probabilistic—in the risky trials—or delayed—in the intertemporal trials) and an alternative option of EUR 5 (this was always the same across all trials and therefore not shown on the screen)

In delay blocks, the variable option was a later-larger (LL) option with either a precise delay or an ambiguous delay range; the alternative option was always the hidden €5 with no delay (sooner-smaller option; SS). In probability blocks, the variable option was a risky option with either a precise probability or an ambiguous probability range; again, the hidden €5 served as the safe option. Example of risky and intertemporal choice trials can be found in Fig. 1.

At the beginning of each trial, the mouse cursor was automatically positioned at the bottom-centre of the screen. Two rectangular choice buttons then appeared: the bottom-left button corresponded to the SS/safe option (“€5 now”), and the bottom-right button corresponded to the LL/risky option (variable reward with either an exact or ambiguous delay/probability). Participants moved the cursor from the centre to click their preferred button; they could revise their selection at any time before confirmation by clicking the opposite button. When ready, they clicked a centrally located “Confirm” button to submit their final choice and advance to the next trial. Reaction time (RT) was defined as the interval from trial onset until the first mouse-click on one of the two choice buttons (rather than the confirm click).

### Intertemporal choices with and without time ambiguity

The intertemporal choice trials included two types of LL options (as mentioned above, the SS option was always €5 now for sure): In the nonambiguous trials, the LL was time-exact (i.e., the delivery time was known exactly). In the ambiguous trials, the LL was time-ambiguous (i.e., the precise delivery time was not known exactly, but instead a range of days was given in which the option would be delivered). There were seven levels of the LL reward amount: €8, €15, €22, €29, €36, €43, or €50. For the *time-exact* LL option, the delay time had nine levels: 10, 26, 50, 76, 100, 150, 174, or 190 days. For the *time-ambiguous* LL option, the ambiguity had five levels (the midpoint of the ambiguous delay range was always 100 days): exactly 100 days (i.e., a range of 0 days and thus no ambiguity), 76–124 days (a range of 48 days), 50–150 days (a range of 100 days), 26–174 days (a range of 148 days), or 0–200 days (a range of 200 days). Thus, there were in total [7 (amount) $\times$ 9 (time-exact delays)] + [7 (amount) $\times$ 5 (time-ambiguous delays)] = 98 unique intertemporal choice trials.

### Risky choices with and without probability ambiguity

In the risky choice task, there were no delays but, in each trial, the option presented on the screen was an uncertain option (i.e., with a probabilistic outcome): In each trial, participants chose between the safe option (always €5 now for sure, corresponding to the SS option in the intertemporal trials) and an uncertain option with a larger reward amount. This uncertain option was either risky (i.e., the probabilities were exactly known) or ambiguous (the probabilities were partially or completely unknown). The reward amounts for the uncertain option were identical to the LL options in the intertemporal choice task. In the *risky* trials, the win probability was either 5%, 13%, 25%, 38%, 50%, 62%, 75%, 87%, or 95%. In the *probability-ambiguity* trials, the probabilities were given as a range—namely, either 50% (a range of 0%, i.e., no ambiguity), 38–62% (a range of 24%), 25–75% (a range of 50%), 13–87% (a range of 74%), or 0–100% (a range of 100%). Thus, like for the intertemporal choice trials, there were in total 98 unique risky choice trials: 7 × 9 = 63 exact trials + 7 × 5 = 35 ambiguous trials.

### Catch trials

To assess whether participants paid attention, 14 catch trials ($1$ per block) were included, where the delayed or probabilistic option did not have a larger amount, but the same amount as the alternative constant option (€5). Therefore, participants who were paying attention would be expected to always choose the alternative constant option in these trials.

### Data exclusion

For all analyses (both the GLMM and the DDM analyses), participants with two or more errors on the catch trials were excluded (this reduced the sample size by 36 to *n* = 1,092 participants). Trials with RTs shorter than 300 ms and longer than 10 s were marked as outlier trials (on average, 7.9% trials for each participant). These outlier trials were excluded from both the DDM and GLMM analyses.

Since online experiments can be vulnerable to low data quality issues, and since these can negatively impact particularly the DDM analyses, which are highly sensitive to RT outliers (Lerche et al., 2017; Peters & D’Esposito, 2020; Ratcliff & Tuerlinckx, 2002), the DDM analyses comprised additional exclusion criteria to ensure the robustness of the parameter estimation in these models. First, participants with 10% or more outlier trials were excluded from the DDM analyses. This criterion excluded 237 participants. Furthermore, those who changed their preferences twice or more per trial (which means that over the whole task, they switched their preference 100 times or more) were also excluded. This criterion led to the exclusion of an additional 186 participants (this exclusion criterion applied only to the DDM analysis (i.e., these data were kept in the GLMM analyses). Trials involving preference switches were deemed unsuitable for our DDM analyses because they indicate multiple stages of evidence accumulation (Pleskac & Busemeyer, 2010). After application of all exclusion criteria, the final sample size for the DDM analyses was 669 participants with 125,375 trials in total (i.e., 187 trials on average per participant). To investigate whether the reduced sample size of the DDM analysis may have led to different results, we re-ran our main GLMM analysis (*n* = 1,092) with the smaller sample (*n* = 669) used in the DDM analysis. The results from the two samples did not exhibit any qualitative differences, and we therefore report the GLMM results from the larger sample in the main text (for completeness, online Supplement A reports the GLMM results from the smaller sample). We present the results of the GLMM for 1,092 samples in the main text’s results section. Due to word count limitations, we provide only a summary of the results there; detailed regression coefficients for this sample are available in Supplement B.

### Data analysis: GLMM

For the regression-type analyses, we ran Bayesian GLMMs using the Python package *Bambi* (Capretto et al., 2022) to analyze both choice and RT data. The choice data were modeled using a logistic model with a Bernoulli distribution. The RT data were modeled using a Wald distribution because the RT data were right skewed (Anders et al., 2016; Farrell & Ludwig, 2008; Heathcote et al., 2002; Lindeløv, 2019). In what follows, we will refer to these two analyses as the choice model and the RT model, respectively.

To study the association between time-ambiguity and probability-ambiguity preferences, we employed separate GLMM that treated RT and choice data from the four distinct types of trials (delay domain/probability domain × exact/ambiguous trials) as dependent variables (DVs). Each GLMM contained two attributes (for exact delay trials, the two attributes were reward amount and delay time; for the ambiguous delay trials, they were reward amount and time ambiguity; for the exact risky trials, they were reward amount and probability; for the ambiguous risky trials, they were reward amount and probability ambiguity) and their interaction as predictors (for exact trials, the interaction term is delay/probability × reward, and for ambiguous trials, the interaction term is ambiguity × reward).

We standardized all independent variables to make their regression coefficients comparable. To keep Type I errors nominal, we adopted a fully maximal model with respect to the random effects (Barr et al., 2013), which treated all fixed effects as random slopes varying over participants. We then extracted the random effects of the regression coefficients (i.e., the BLUPs) for both time ambiguity and probability ambiguity. To quantify the association between individual differences in time-ambiguity and probability-ambiguity preferences, we then computed a Bayesian robust correlation between these regression coefficients using the Python package Pymc (Abril-Pla et al., 2023). This robust correlation approach utilizes the *t* distribution instead of the Gaussian distribution, making it less sensitive to outliers (Lange et al., 1989; McElreath, 2020; Wilcox, 2011). 

Bayesian Region of Practical Equivalence (ROPE) tests were used to assess statistical significance of all effects of interest (Kruschke, 2013, 2015). The ROPE is a prespecified range of values, typically centered around a null value, within which parameters or models are considered practically equivalent to zero. The ROPE + HDI decision rule (Kruschke, 2018, 2021) assesses the practical significance of findings by comparing the highest density interval (HDI) of parameter estimates with a prespecified ROPE. If the entire HDI falls within the ROPE, it suggests that the difference from zero is not practically significant. If the HDI falls completely outside the ROPE, it indicates a practically significant difference from zero. If the HDI partially overlaps with the ROPE, the evidence is inconclusive.

### Data analysis: DDM

We jointly modeled the choice and RT data using the drift-diffusion model (DDM), a type of decision-making model of the class of sequential sampling models (SSMs; Gold & Shadlen, 2007; Ratcliff et al., 2016). SSMs characterize decision-making as a noisy, bounded evidence-accumulation process, where a response is generated only once the accumulated evidence meets or exceeds a specific criterion. The decision time is the period required for evidence accumulation. The canonical DDM dissects RT and choice data into four meaningful cognitive parameters: speed of evidence integration (drift rate: $v$), decision boundary ($A$), starting point of evidence integration ($z$), and the non-decision time parameter (${t}_{0}$) which governs visual encoding and motor time. The drift rate reflects how individuals perceive and integrate stimulus information—specifically reward magnitude and delay/probability attributes—into the decision‐making process. It is modulated by participants’ decision strategies (Le Houcq Corbi & Soutschek, 2024; Zhao et al., 2019). And attentional allocation across attributes or options playing a central role in shaping the drift rate (Gluth et al., 2018, 2020; Krajbich et al., 2010). The decision boundary defines the required evidence-level for making a decision, reflecting response caution and speed–accuracy trade-offs (Bogacz et al., 2006, 2010; Cavanagh et al., 2011; Forstmann et al., 2008). The starting point represents the initial bias or predisposition towards one decision alternative over another before any evidence is considered (Chen & Krajbich, 2018; Chen et al., 2024; Desai & Krajbich, 2021; Zhao et al., 2019).

In line with previous DDM applications in preferential decision-making tasks, we assumed that participants would choose the variable option (e.g., the LL option in the intertemporal trials and the risky option in the risky trials) if the accumulated evidence reaches the upper boundary, and they would select the constant option (i.e., SS/safe option) if the evidence reaches the lower boundary (Amasino et al., 2019; Diederich & Trueblood, 2018; Fontanesi et al., 2019; Glickman & Usher, 2019; Peters & D’Esposito, 2020; Smith & Peters, 2022).

Research on risky and intertemporal decision-making has increasingly applied the diffusion model to analyze response time (RT) and choice data. For instance, Diederich and Trueblood (2018) developed a two-stage diffusion model, assuming that the drift rate is determined by the utility calculated using prospect theory. Following this approach, we also assumed that the drift rate is determined by the subjective value (SV) difference between the two options, where SV is computed using the utility function. The drift rate is calculated as

1 $$v=\eta \times \left(S{V}_{LL,Risky}-S{V}_{SS,Safe}\right),$$

where $\eta$ is a scaling parameter that ensures that the speed of evidence accumulation falls within the proper range (Gluth et al., 2015; Pedersen et al., 2017). Therefore, the larger the SV difference, the faster and the more likely the option with the larger SV is chosen (Busemeyer & Townsend, 1993; Krajbich et al., 2010).

For trials with only one response, we model the RT and choice data using the Wiener first passage time:

2 $$R{T}_{i}, Choic{e}_{i}\sim WFTP\left(A,{t}_{0},z,{v}_{i}\right).$$

In trials where participants switched their preference at least once, the RT data are not suitable for DDM. Consequently, in these trials, we exclusively employed the choice function of DDM to model the choice, but not the RT data (Bogacz et al., 2006; Miletić et al., 2020):

3 $$P\left(LL/Risky\right)=\frac{{e}^{-2\times A\times z\times {v}_{t}}-1}{{e}^{-2\times A\times {v}_{t}}-1}.$$

### Intertemporal choice models

Intertemporal choices have traditionally been modeled using discounting models, such as the hyperbolic or exponential discounting models (Figner et al., 2010; Johnson & Bickel, 2002; Kable & Glimcher, 2007; Loewenstein & Prelec, 1992). However, recent advances in the application of the DDM to intertemporal choice data have shown that simple attribute-wise models often fit the data better than hyperbolic models (Amasino et al., 2019; Ballard et al., 2023; Dai & Busemeyer, 2014; Soutschek & Tobler, 2023; Zhao et al., 2019). This result is consistent with earlier choice-based studies that identified the attribute-wise model as the better model in intertemporal choice (Cheng & González-Vallejo, 2016; Ericson et al., 2015; He et al., 2023; Peters & D’Esposito, 2016; Read et al., 2013; Scholten & Read, 2010). In the current study, we therefore compared several variants of hyperbolic and attribute-wise models with capture the intertemporal decisions across both time-ambiguous and time-exact trials. As benchmarks, we also included the standard one-parameter hyperbolic model and the two-parameter general hyperbolic model (both without incorporating ambiguity) in the modeling analysis. Details of the intertemporal choice model formulas can be seen in Table 1.

**Table 1:** Drift-rate function of all diffusion models fitted to the intertemporal choice data

Models	Drift-rate function
1. Hyperbolic	$\text{v}=\upeta \times (\frac{{\text{V}}_{\text{LL}}}{1+\text{k}\times {\text{D}}_{\text{LL}}}-\frac{{\text{V}}_{\text{LL}}}{1+\text{k}\times {\text{D}}_{\text{SS}}})$
2. Hyperbolic + additive model	$\text{v}=\upeta \times (\frac{{\text{V}}_{\text{LL}}}{1+\text{k}\times {\text{D}}_{\text{LL}}}-\frac{{\text{V}}_{\text{LL}}}{1+\text{k}\times {\text{D}}_{\text{SS}}}-{\upbeta }_{\text{ITC}}\times \frac{{\text{Amb}}_{\text{ITC}}}{2})$
3. Hyperbolic + time perception model	$\text{v}=\upeta \times (\frac{{\text{V}}_{\text{LL}}}{1+\text{k}\times {(\text{D}}_{\text{LL}}-{\upbeta }_{\text{ITC}}\times {\text{Amb}}_{\text{ITC}})}-\frac{{\text{V}}_{\text{LL}}}{1+\text{k}\times {\text{D}}_{\text{SS}}})$
4. Generalized hyperbolic model	$\text{v}=\upeta \times (\frac{{\text{V}}_{\text{LL}}}{1+{\left(\text{k}\times {\text{D}}_{\text{LL}}\right)}^{\text{s}}}-\frac{{\text{V}}_{\text{LL}}}{1+{\left(\text{k}\times {\text{D}}_{\text{SS}}\right)}^{\text{s}}})$
5. Generalized hyperbolic model + additive model	$\text{v}=\upeta \times (\frac{{\text{V}}_{\text{LL}}}{1+{\left(\text{k}\times {\text{D}}_{\text{LL}}\right)}^{\text{s}}}-\frac{{\text{V}}_{\text{LL}}}{1+{\left(\text{k}\times {\text{D}}_{\text{SS}}\right)}^{\text{s}}}-{\upbeta }_{\text{ITC}}\times \frac{{\text{Amb}}_{\text{ITC}}}{2})$
6. Generalized hyperbolic model + time perception model	$\text{v}=\upeta \times (\frac{{\text{V}}_{\text{LL}}}{1+{\left(\text{k}\times {\text{D}}_{\text{LL}}-{\upbeta }_{\text{ITC}}\times \frac{{\text{Amb}}_{\text{ITC}}}{2}\right)}^{\text{s}}}-\frac{{\text{V}}_{\text{LL}}}{1+{\left(\text{k}\times {\text{D}}_{\text{SS}}\right)}^{\text{s}}})$
7. Attribute-wise model	$\text{v}={\text{w}}_{\text{r}}\times \left({\text{V}}_{\text{LL}}-{\text{V}}_{\text{SS}}\right)+{\text{w}}_{\text{t}}\times {(\text{D}}_{\text{LL}}-{\text{D}}_{\text{SS}})$
8. Attribute-wise model + interaction between reward and delay	${\text{w}}_{\text{r}}\times \left({\text{V}}_{\text{LL}}-{\text{V}}_{\text{SS}}\right)+{\text{w}}_{\text{t}}\times {(\text{D}}_{\text{LL}}-{\text{D}}_{\text{SS}})+{\text{w}}_{\text{inter}}({\text{V}}_{\text{LL}}-{\text{V}}_{\text{SS}})\times ({\text{D}}_{\text{LL}}-{\text{D}}_{\text{SS}})$
9. Attribute-wise model + ambiguity	$\text{v}={\text{w}}_{\text{r}}\times \left({\text{V}}_{\text{LL}}-{\text{V}}_{\text{SS}}\right)+{\text{w}}_{\text{t}}\times {(\text{D}}_{\text{LL}}-{\text{D}}_{\text{SS}})+{\text{w}}_{{\text{amb}}_{\text{ITC}}}\times \frac{{\text{Amb}}_{\text{ITC}}}{2}$
10. Attribute-wise model + ambiguity + interaction between reward and delay	$\text{v}={\text{w}}_{\text{r}}\times \left({\text{V}}_{\text{LL}}-{\text{V}}_{\text{SS}}\right)+{\text{w}}_{\text{t}}\times {(\text{D}}_{\text{LL}}-{\text{D}}_{\text{SS}})+{\text{w}}_{\text{inter}}({\text{V}}_{\text{LL}}-{\text{V}}_{\text{SS}})\times ({\text{D}}_{\text{LL}}-{\text{D}}_{\text{SS}})+{\text{w}}_{{\text{amb}}_{\text{ITC}}}\times \frac{{\text{Amb}}_{\text{ITC}}}{2}$
11. Attribute-wise model + ambiguity + interaction between reward and ambiguity	$\text{v}={\text{w}}_{\text{r}}\times \left({\text{V}}_{\text{LL}}-{\text{V}}_{\text{SS}}\right)+{\text{w}}_{\text{t}}\times {(\text{D}}_{\text{LL}}-{\text{D}}_{\text{SS}})+{\text{w}}_{{\text{amb}}_{\text{ITC}}}\times {\text{Amb}}_{\text{ITC}}+{\text{w}}_{{\text{inter}}_{\text{amb}}}({\text{V}}_{\text{LL}}-{\text{V}}_{\text{SS}})\times \frac{{\text{Amb}}_{\text{ITC}}}{2}$
12. Attribute-wise model + ambiguity + interaction between reward and ambiguity + interaction between reward and delay	$\text{v}={\text{w}}_{\text{r}}\times \left({\text{V}}_{\text{LL}}-{\text{V}}_{\text{SS}}\right)+{\text{w}}_{\text{t}}\times {(\text{D}}_{\text{LL}}-{\text{D}}_{\text{SS}})+{\text{w}}_{{\text{amb}}_{\text{ITC}}}\times {\text{Amb}}_{\text{ITC}}+{\text{w}}_{\text{inter}}({\text{V}}_{\text{LL}}-{\text{V}}_{\text{SS}})\times ({\text{D}}_{\text{LL}}-{\text{D}}_{\text{SS}})+{\text{w}}_{{\text{inter}}_{\text{amb}}}({\text{V}}_{\text{LL}}-{\text{V}}_{\text{SS}})\times \frac{{\text{Amb}}_{\text{ITC}}}{2}$

### Risky choice models

To model decisions involving probability ambiguity, the most often used quantitative model is the Gilboa–Schmeidler (G-S) model which posits that probability-ambiguity influences the perceived probability (Gilboa & Schmeidler, 1989; Levy et al., 2010). Apart from this model, since we wanted to make the model comparison matrix as symmetrical as possible across the time and probability domains, we also included an additive model among the risky choice models, which we call the expected utility (EU) + additive ambiguity model.

In total, the candidate models in our risky choice analysis include the G-S model, the expected utility model, the expected utility + additive ambiguity model, and the risky choice version of the previously mentioned attribute-wise models in intertemporal choice. Details of the formulas can be seen in Table 2.

**Table 2:** Drift-rate function of all diffusion models fitted to the risky choice data

Models	Drift-rate function
1. Expected utility model	$v=\eta \times ({P}_{risky}\times {V}_{risky}^{\alpha }-{{P}_{sure}\times V}_{sure}^{\alpha })$
2. Gilboa–Schmeidler model	$v=\eta \times ({(P}_{risky}-{\beta }_{risk}\times \frac{Am{b}_{risk}}{2})\times {V}_{risky}^{\alpha }-{P}_{sure}\times {V}_{sure}^{\alpha })$
3. Expected utility + additive model	$v=\eta \times ({P}_{risky}\times {V}_{risky}^{\alpha }-{P}_{sure}\times {V}_{sure}^{\alpha }-{\beta }_{risk}\times \frac{Am{b}_{risk}}{2})$
3. Attribute-wise model	$v={w}_{r}\times \left({V}_{risky}-{V}_{safe}\right)+{w}_{p}\times {(P}_{risky}-{P}_{safe})$
4. Attribute-wise model + interaction between reward and probability	$v={w}_{r}\times \left({V}_{risky}-{V}_{safe}\right)+{w}_{p}\times {(P}_{risky}-{P}_{safe})+{w}_{inter}({V}_{risk}-{V}_{safe})\times ({P}_{risky}-{P}_{safe})$
5. Attribute-wise model with ambiguity	$v={w}_{r}\times \left({V}_{risky}-{V}_{safe}\right)+{w}_{p}\times {(P}_{risky}-{P}_{safe}) +{w}_{am{b}_{risky}}\times \frac{Am{b}_{ITC}}{2}$
6. Attribute-wise model with ambiguity with interaction between reward and probability	$v={w}_{r}\times \left({V}_{risky}-{V}_{safe}\right)+{w}_{p}\times {(P}_{risky}-{P}_{safe}) +{w}_{am{b}_{risky}}\times Am{b}_{risky}+{w}_{inter}({V}_{risk}-{V}_{safe})\times ({P}_{risky}-{P}_{safe})$
7. Attribute-wise model + ambiguity + interaction between reward and ambiguity	$v={w}_{r}\times \left({V}_{risky}-{V}_{safe}\right)+{w}_{p}\times {(P}_{risky}-{P}_{safe})+{w}_{am{b}_{ITC}}\times Am{b}_{risky}+{w}_{inte{r}_{amb}}({V}_{risk}-{V}_{safe})\times \frac{Am{b}_{risky}}{2}$
8. Attribute-wise model + ambiguity + interaction between reward and ambiguity + interaction between reward and probability	$v={w}_{r}\times \left({V}_{risky}-{V}_{safe}\right)+{w}_{p}\times {(P}_{risky}-{P}_{safe})+{w}_{am{b}_{ITC}}\times Am{b}_{risky}+{w}_{inter}({V}_{risk}-{V}_{safe})\times ({P}_{risky}-{P}_{safe})+{w}_{inte{r}_{amb}}({V}_{risk}-{V}_{safe})\times \frac{Am{b}_{risky}}{2}$

### Model fitting and model comparison

All models were fitted using a Bayesian approach with the probabilistic language Stan (Carpenter et al., 2017). We used a normal distribution with a large standard deviation to mimic a uniform distribution prior for all parameters. The model fitting was performed using two MCMC chains with CmdStanPy (van Ravenzwaaij et al., 2018). Each chain consisted of 4,500 iterations after 3,500 initial warm-up iterations. Model convergence was confirmed by checking the Gelman–Rubin R̂ statistics for each parameter (R̂ < 1.005 for all parameters; Gelman & Rubin, 1992).

Model comparison was performed using the Pareto smoothed importance sampling–leave-one-out cross validation (PSIS–Loo) with the Python package ArivZ (Kumar et al., 2019). Individual PSIS–Loo values were calculated and then used for a random-effect Bayesian model selection (RE-BMS), which computes the protected exceedance probability (PXP) of each model, representing the belief that one model is more likely to be the best model than all other candidate models (Acerbi et al., 2018; Penny, 2012; Rigoux et al., 2014; Stephan et al., 2009). If a model has a PXP > 0.95, this is considered significant evidence for supporting the model (Correa et al., 2018; Iglesias et al., 2013).

We then performed a parameter recovery for the best-fitting model identified by the PSIS–Loo: Simulated data were generated using parameters estimated from each participant, which were then fitted to the model. Parameter identification was assessed by correlating the fitted parameters with those used in the simulation (Danwitz et al., 2022; Lerche et al., 2017; McDougle & Collins, 2020; Wilson & Collins, 2019). Details of parameter recovery can be found in Supplement D. Most parameter correlation coefficients were larger than 0.7, indicating that the parameters were reliably estimated.

However, it has been argued that the PSIS–Loo has the problem that it tends to favor more complex models (Gronau & Wagenmakers, 2019; Lu et al., 2017). Accordingly, to supplement our model comparison findings, we also computed the log-model evidence (LME) using kernel density estimation (KDE) as an alternative metric for model comparisons (Bos, 2002; Llorente et al., 2023). Since model comparisons using LME and PSIS–Loo yielded analogous results in our case, we primarily present the PSIS–Loo findings in the main text, and report the LME results in Supplement E.

The earlier-mentioned model comparisons aim to select one single best-fitting model. However, this approach may suffer from the uncertainty of model comparison metrics. In contrast, multimodel inference approaches like Bayesian model averaging (BMA) offer advantages over single-model comparisons by also considering the model uncertainty (Berger & Molina, 2005; Boehm et al., 2023; Clyde, 2000; Clyde et al., 2011; Hinne et al., 2020; Hoeting, 2002).

One specific application of BMA is the Inclusion Bayes factor ($B{F}_{inclusion}$), which evaluates the necessity of a specific variable within a model (Boehm et al., 2023). $B{F}_{inclusion}$ allows us to compare one type of model with another type of model. In our case, we can use $B{F}_{inclusion}$ to compare several types of ambiguity models against several types of nonambiguity models at the same time in one single comparison.

The $B{F}_{inclusion}$ is based on the model evidence, whereas metrics like AIC or PSIS–Loo can be used for computing the *Pseudo* Inclusion Bayes Factor ($PB{F}_{inclusion}$) which mimics the $B{F}_{inclusion}$. To compute $PB{F}_{inclusion}$, an Akaike-type of model weight is needed, which is computed using a *softmax* function. For details of the computation of $PB{F}_{inclusion}$, see Supplement F.

To maintain compatibility with the single-model inferences, we only report $PB{F}_{inclusion}$ in the main text, while including $B{F}_{inclusion}$ in Supplement F. We leveraged $PB{F}_{inclusion}$ to formally compare the group of ambiguity models against the group of nonambiguity models, as well as the group of attribute-wise models against the group of integrated-value models. Similarly, like the single-model comparisons, $PB{F}_{inclusion}$ was then used to compute RE-BMS for group-level comparisons.

## Results

### GLMM results

**Fig. 2:**
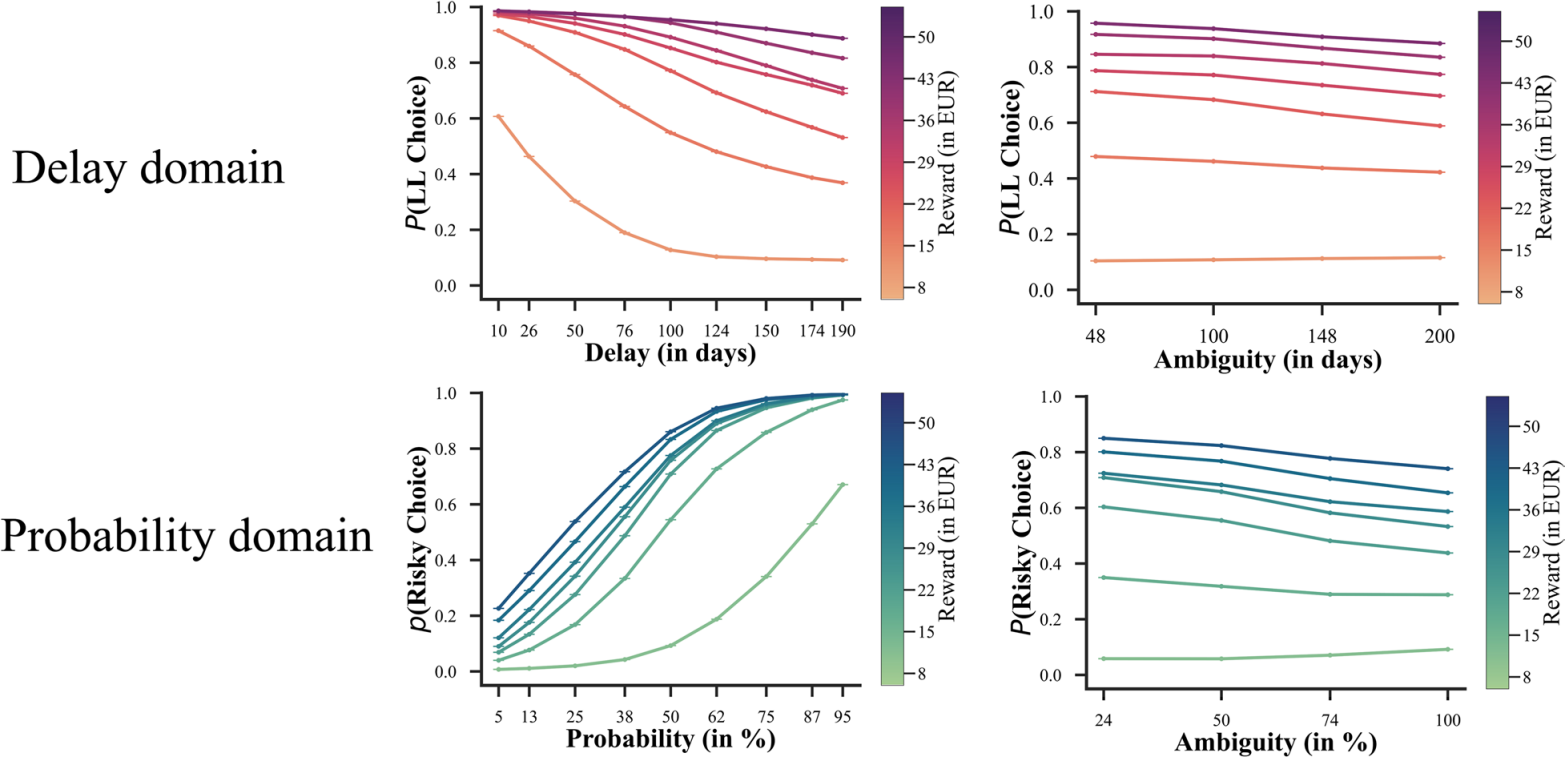
Choice data of risky and intertemporal choice tasks. *Note.* We partitioned our task trials into two orthogonal dimensions. The first dimension categorizes trials based on the delay versus probability domain. The second dimension distinguishes between exact versus ambiguous trials. In the delay domain, participants chose the later-larger option more frequently withthe increasing of reward and the decreasing of delay time and ambiguity (in days). In the probability domain, participants chose the risky option more frequently with the increasing of reward and gain probability and the decreasing of ambiguity (in %)

**Fig. 3:**
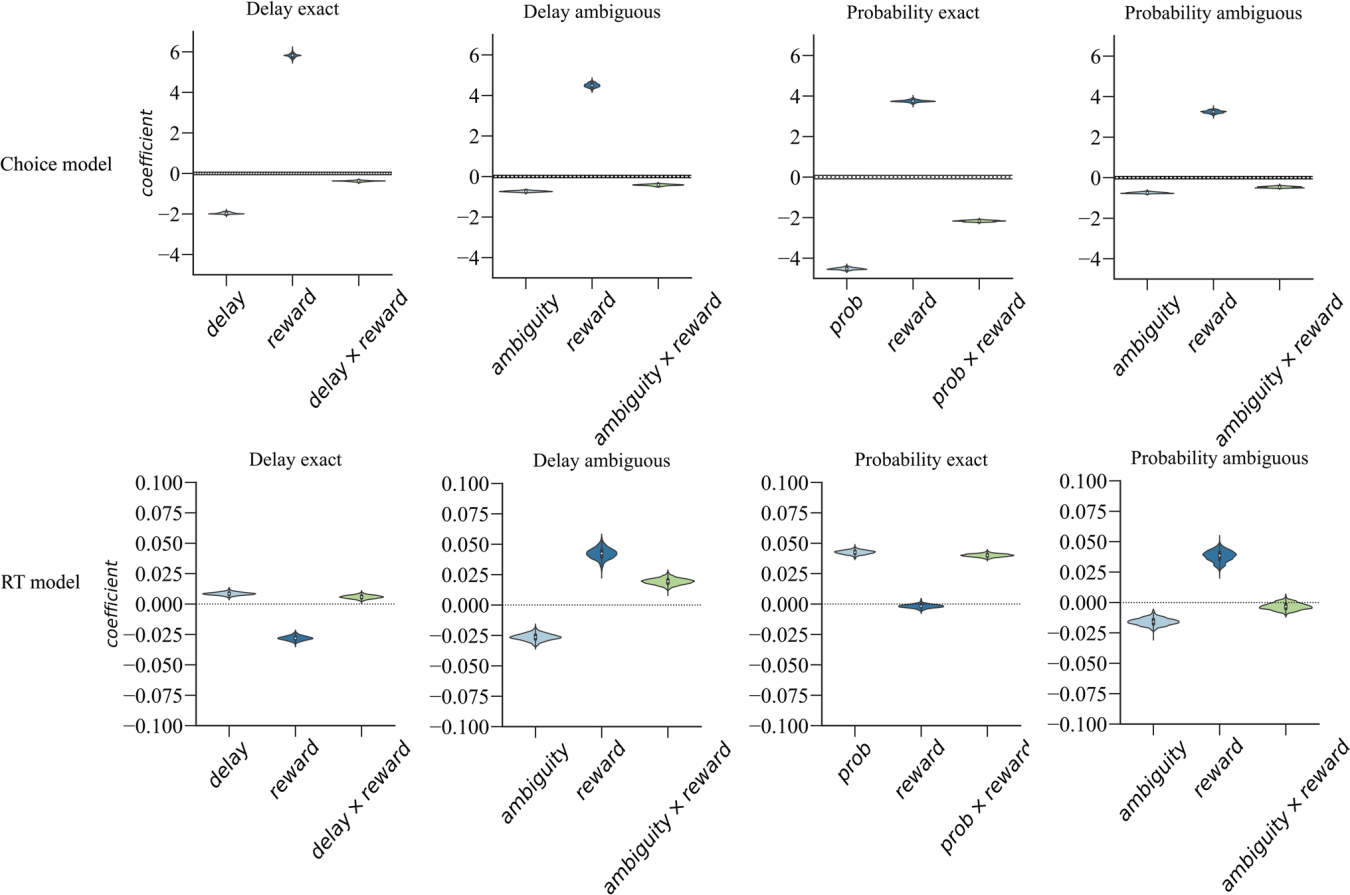
Results of the Bayesian mixed-effects models of choice and RT. *Note.* The x-axis represents the different predictors, the y-axis represents the posterior distribution with the violin plots showing the 95% HDI of the respective fixed-effect regression coefficient. Note that we standardized all independent variables to make their regression coefficients comparable. The ROPE range for the choice models is demarcated by the horizontal lines centered around 0 (they are very close together, given the impression of a single line). The ROPE range for the RT model is not depicted on the plots as it is disproportionately larger than the distribution of the coefficient, rendering it unsuitable for a scaled visual representation (i.e., the ROPE range is outside of the range depicted on the RT y-axes). For all choice models, the ROPE ranges are ±0.06. Among the RT models, the ROPE range varies: ±3.33 for the exact delay trials, ±4.55 for the ambiguous delay trials, ±3.04 for the exact probability trials, and ±4.72 for the ambiguous probability trials. A regression coefficient is deemed significant if its posterior distribution falls completely outside of the ROPE range. The 95% HDIs of all predictors of the choice models fell outside of the ROPE range while those of the RT models all fell within a ±0.1 range and thus were clearly within the ROPEs, meaning that all the predictors of the choice models were significant while all the predictors of the RT models were nonsignificant and practically equivalent to 0

Choice data of risky and intertemporal choice tasks can be found in Fig. 2. The mixed-effects choice model results are presented in Fig. 3. During exact trials, we observed that increasing the reward magnitude of the LL [risky] choice option led to an increasing preference for the LL [risky] option (time domain: $\beta$= 5.81, 95% HDI [5.58, 5.99]; probability domain: $\beta$= 3.75, 95% HDI [3.63, 3.87]). Conversely, longer delays and lower gain probabilities decreased preference for these options (time: $\beta$= −1.97, 95% HDI = [−2.04, −1.87]); probability: $\beta$= −4.52, 95% HDI [−4.62, −4.40]). The interaction terms between reward magnitude and (i) delay duration in the intertemporal task and (ii) probability in the risky task were both significant (time: $\beta$= −0.38, 95% HDI [−0.43, −0.32]; probability: $\beta$= −2.17, 95% HDI [−2.23, −2.01]), indicating that both the effects of delay duration and probability became weaker with increasing reward magnitudes (see Fig. 3, top panels).

In ambiguous trials, an increase in reward magnitude also led participants to choose LL options ($\beta$= 4.50, 95% HDI [4.28, 4.68]) and risky options more frequently ($\beta$= 3.25, 95% HDI [3.12, 3.38]). Moreover, we observed that participants exhibited ambiguity aversion in both the time ($\beta$= −0.73, 95% HDI [−0.80, −0.67]) and the probability domains ($\beta$= −0.75, 95% HDI [−0.80, −0.67]), with comparable magnitudes for the regression coefficients across domains. Consistent with the exact trials, the interaction terms between reward magnitude and ambiguity were significant in both domains, indicating that ambiguity aversion decreased as reward magnitudes increased (time: $\beta$= −0.42, 95% HDI [−0.49, −0.35]; probability: $\beta$= −0.46, 95% HDI [−0.52, −0.40]; see Fig. 3, bottom panels). Importantly, for all predictors their full posterior distribution fell completely outside of the ROPE range, indicating that all the observed effects were significant.

In the RT analysis, we found that the 95% HDI of some effects did not include zero; however, the values of these regression coefficients were small, and all regression coefficients’ full posterior distributions fell completely within their ROPE ranges. These results indicate that variations in delay duration, gain probabilities, ambiguity levels, and amounts had minimal impact on the RTs. Further details of the RT model results are provided in Supplement B.

To explore the strength of the association between individuals’ preferences for ambiguity across the probability and time domains, we calculated a robust correlation coefficient using the random effect ambiguity regression coefficients for each participant. The correlation coefficient indicated a relatively small but significant association between individuals’ time-ambiguity and probability-ambiguity coefficients of $r$ = 0.16 (95%, HDI [0.01, 0.23]). The correlation’s full posterior distribution fell completely outside of the ROPE (see Fig. 5C, D), indicating that individuals who show ambiguity aversion in the time domain are also significantly more likely to show ambiguity aversion in the probability domain, and vice versa.

### Drift-diffusion model results

Consistent with previous findings in the delay domain, attribute-wise models outperformed hyperbolic discounting models (Amasino et al., 2019; Dai & Busemeyer, 2014). Specifically, the RE-BMS results favored the attribute-wise model that incorporated ambiguity and a reward × delay time interaction as the best fitting model (PXP = 1). We additionally performed a RE-BMS analysis that only included the best-fitting attribute-wise model and two best-fitting hyperbolic models (the time perception model and the additive model):^1^ The result confirmed that the attribute-wise model outperformed the two hyperbolic models (PXP = 1). This suggests that the attribute-wise model provides a more precise and comprehensive representation of the cognitive processes and decision-making mechanisms underpinning ambiguity-aversion in intertemporal choice (see Tables 3 and 4 for detailed information). By examining the sign of the weight parameter for ambiguity in the attribute-wise model that was fitted per participant, we can observe that the majority of participants (469 out of 669) exhibited ambiguity aversion in the delay domain, consistent with Ikink et al. (2019, 2023). Summary statistics of the best-fitting model parameters can be found in Supplement G.

**Table 3:** Model comparison based on PSIS–Loo for the intertemporal choice task (ordered by median PSIS–Loo, i.e., from best- to least-fitting model)

	PSIS–Loo (Median)	PXP
Attribute-wise model + ambiguity + interaction between reward and delay	−178.65	1
Generalized hyperbolic + additive model	−179.02	0
Attribute-wise model + ambiguity + interaction between reward and ambiguity	−179.84	0
Generalized hyperbolic + time-perception model	−179.11	0
Attribute-wise model	−180.38	0
Attribute-wise model + interaction between reward and delay	−180.38	0
Attribute-wise model + ambiguity	−180.46	0
Generalized hyperbolic model	−180.57	0
Attribute-wise model + interaction between reward and delay + interaction between reward and ambiguity	−181.46	0
Additive model	−183.14	0
Time-perception model	−184.29	0
Hyperbolic model	−191.46	0

**Table 4:** Model comparison based on PSIS–Loo for the risky choice task (ordered by median PSIS–Loo, i.e., from best- to least-fitting model)

	PSIS–Loo (Median)	PXP
Gilboa-Schmeidler model	−172.61	1
Expected utility + additive model	−172.70	0
Attribute-wise model + interaction between reward and probability	−180.46	0
Expected utility model	−181.62	0
Attribute-wise model with ambiguity with interaction between reward and probability	−181.63	0
Attribute-wise model with ambiguity	−183.88	0
Attribute-wise model with ambiguity with interaction between reward and ambiguity	195.77	0
Attribute-wise model	−199.88	0
Attribute-wise model with ambiguity with interaction between reward and ambiguity with interaction between reward and probability	−199.93	0

In the probability domain, in contrast, no clear winning model emerged: The classical G-S model displayed nearly identical PSIS–Loo values as the EU + additive model. While the RE-BMS analysis that included all models supported the G-S model (PXP = 1), the RE-BMS analysis comparing only these two models indicated no clear winner (G-S model, PXP = 0.52; expected utility + additive model, PXP = 0.48). With the current experimental design, it appears therefore impossible to differentiate between two models.

Next, we examined the ambiguity aversion parameter in each of the two best-fitting probability-ambiguity models and found that they are strongly correlated (mean $r=$.94, 95% HDI [.93,.95]). This suggests that despite differences in their utility functions, the ambiguity preferences detected by the two models are highly similar.

**Fig. 4:**
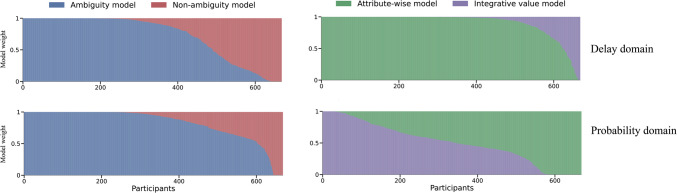
Model weight across participants. *Note.* Akaike-type of model weight (computed with PSIS-Loo) per participant for the ambiguity model and the non-ambiguity model. Of the 669 participants, 583 (87%) demonstrated model weights greater than 0.5, favoring the ambiguity model over the non-ambiguity model in the delay domain. In the probability domain, 558 (83%) participants'model weights supported the ambiguity model over the non-ambiguity model

Single-model inference may be impacted by model misspecification, whereas model averaging can mitigate the effects of such misspecification. Consequently, we utilized model averaging to formally compare models with and without ambiguity, as well as attribute-wise models versus integrated-value models, by computing the pseudo-inclusion Bayes factor ($PB{F}_{inclusion}$) for each participant. We found that most participants were best explained by models incorporating ambiguity (see Fig. 4). The $PB{F}_{inclusion}$ results showed for the probability domain that there was substantial evidence supporting the models *with* ambiguity (median $PB{F}_{inclusion}=$ 9.87, PXP = 1), over the models without ambiguity. For the intertemporal choices, $PB{F}_{inclusion}$ also indicated support in favor of ambiguity models (median $PB{F}_{inclusion}=$ 2.41, PXP = 1).

Our $PB{F}_{inclusion}$ results revealed a strong preference for the attribute-wise model in the intertemporal choice task (median $PB{F}_{inclusion}=$ 6.63, PXP = 1). Conversely, in the risky choice task, there was support for the integrated-value model (median $PB{F}_{inclusion}=$ 2.02, PXP = 1).

**Fig. 5:**
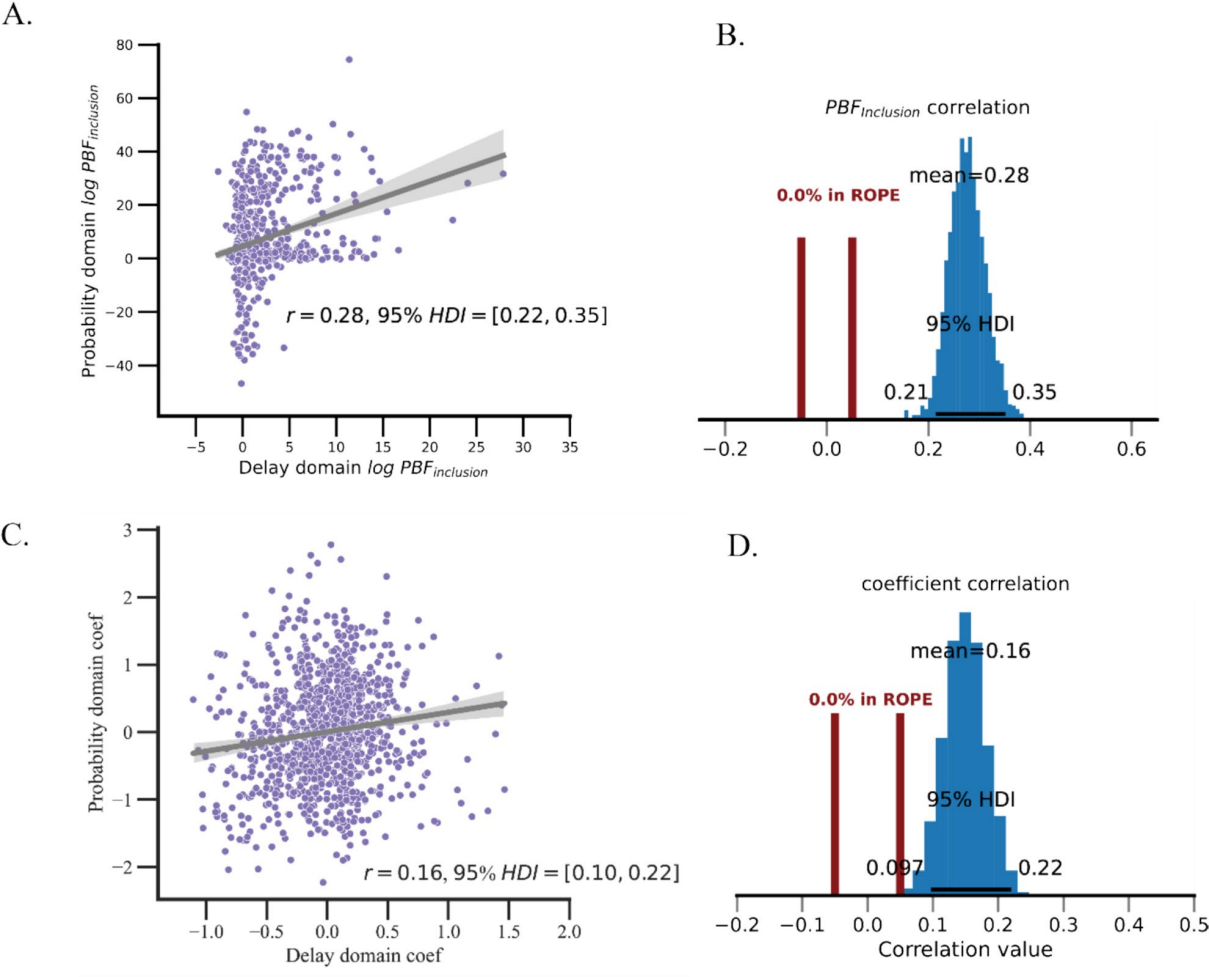
Examining ambiguity preferences across probability and delay domains. *Note.* Panel A shows a scatterplot visualizing the correlation of log *PBFinclusion* in the delay and probability domains, where log *PBFinclusion* is derived from the comparison between the ambiguity and non-ambiguity models. Panel C offers a scatterplot detailing the association between the random effect coefficients (i.e., BLUPs) of ambiguity derived from the linear mixed-effects analysis. Panels B and D show the posterior distribution of the estimated correlation coefficient; in both cases, all samples fall outside of the ROPE range, indicating that the correlations are significant

Since computational models offer more comprehensive interpretations of behavioral data compared to regression analyses, they can serve as a tool for probing individual differences. Accordingly, we computed the correlation between the log $PB{F}_{inclusion}$ from the risky choice and intertemporal choice tasks. In line with the GLMM analysis, we observed a substantial correlation between the log $PB{F}_{inclusion}$ (mean $r=$.28, 5% HDI [.22,.34], 0% HDI in the ROPE range, see Fig. 5A, B). This means that participants who are better fitted with a model that incorporated ambiguity for the delay domain, they were also fitted better by an ambiguity model for the probability domain (and vice versa). We further explored the relationship between ambiguity-preference parameters across the two tasks, with detailed results in Supplement G. In summary, the correlation of parameter values, though slightly lower than the log $PB{F}_{inclusion}$ correlation, remained significant (0% HDI in ROPE range).

**Fig. 6:**
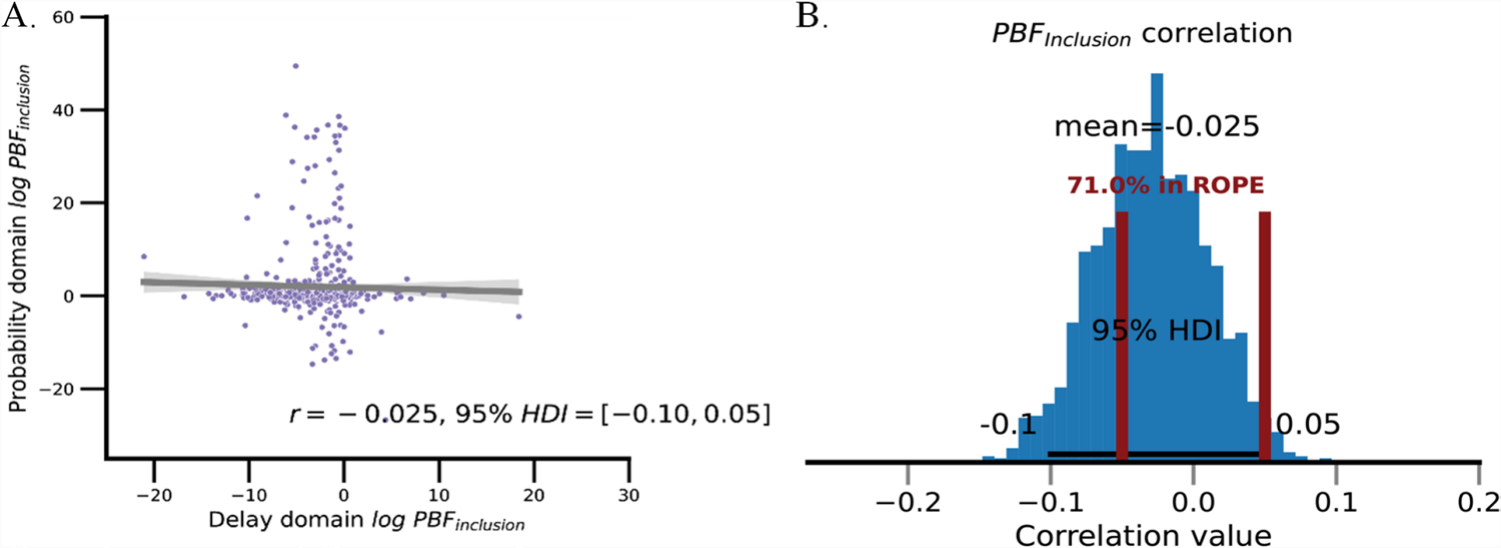
Comparison of the best-fitting models across the probability and delay domains. *Note.* Panel A shows a scatterplot visualizing the correlation of log *PBFinclusion* in the delay and probability domains, where log *PBFinclusion* is derived from the comparison between the attributewise models and integrative-value models. Panel B depicts the posterior distribution of this correlation. As 71% of posterior samples fall within the ROPE range, we interpret this as the absence of a significant correlation

In contrast, when investigating the correlation between the log $PB{F}_{inclusion}$ which aims to compare the attribute-wise model and integrated-value model in the intertemporal choice task and risky choice task, we found no significant relationship between the $PB{F}_{inclusion}$ of the two tasks (mean $r=$ −.03, 95% HDI [−.10,.03], 97.24% HDI is in the ROPE range, see Fig. 6). This suggests that strategies individuals employ in decision-making regarding the integration of different attribute values do not share commonality across various contexts.

## Discussion

The current study analyzed a large dataset to (i) explore the link between time-ambiguity and probability-ambiguity preferences and (ii) find the best computational models describing decision-making in intertemporal and risky choices involving ambiguity. To be able to address these questions, participants were asked to make decisions involving probability ambiguity and decisions involving time ambiguity. Our GLMM results revealed that on average, participants were averse to ambiguity across both the time and the probability domains: As ambiguity levels increased, participants showed an increasing preference for the unambiguous option. These findings were significant using the ROPE decision rule, indicating that the observed effects are practically significant and their statistical significance not merely an artifact of the relatively large sample size. We also found a modest yet significant correlation between ambiguity preferences across domains.

To obtain insight into the cognitive processes underlying participants’ decisions, we fitted several DDMs to the data. By using PSIS–Loo and RE-BMS for model evaluation, we found that for intertemporal choices, an attribute-wise model—more specifically, one which incorporates ambiguity as a separate attribute in addition to time and reward, as well as the interaction between time and reward—outperformed other candidate models, including those identified as best fitting by Ikink et al. (2019). For risky choices, the commonly used G-S model and the EU + additive model—which assumes ambiguity does not interact with the reward, exhibited comparable performance. Both models are integrated-value models and surpassed other models that did not account for the effect of ambiguity.

We employed model averaging to compute $PB{F}_{inclusion}$ as a model comparison metric between ambiguity and nonambiguity models. Analyzing $PB{F}_{inclusion}$ data across the time and probability domains showed a notable correlation. In conjunction with the GLMM results, these results suggest the existence of ambiguity preferences that are partially shared and partially independent across domains (i.e., the observed correlation, relatively moderate in strength but still clearly significant, between ambiguity preferences in the time and probability domains might indicate a general ambiguity aversion, either as an alternative to or alongside domain-specific ambiguity preferences).

A potential explanation for the observed partially domain-independent ambiguity preferences might relate to limited cognitive resources: According to the resource-rational theory of attention and cognitive control, cognitive capacity is limited, and individuals allocate attention based on the costs and benefits of allocating these attention (Lieder et al., 2018; Shenhav et al., 2013; van den Berg & Ma, 2018). If the additional attention does not provide sufficient reward relative to its cost, they are less likely to exert it. While processing ambiguous options—whether in the delay or probability domain—requires greater cognitive effort, it does not necessarily yield proportional rewards, potentially leading individuals to allocate less attention to these options. Consequently, this reduced focus could lower the likelihood of choosing the ambiguous options in both the time and probability domain. Future work could investigate whether this holds also for other decision domains (e.g., social decisions).

Our results also have implications for research on temporal impulsivity: Typically, temporal impulsivity has been measured using intertemporal choice tasks that use exact delays (Ainslie, 1975; Caswell et al., 2015; Herman et al., 2018). However, temporal impulsivity may be influenced by multiple factors, with aversion to time ambiguity being one such factor. This could have implications for how we design interventions to reduce impulsive behavior. For example, impulsive behavior, such as smoking, might be attributable also to an aversion to time ambiguity, rather than a steeper delay discounting curve alone.

In the domain of probability, participants exhibiting more pronounced aversion to probability ambiguity were found to have lower cognitive functions, including attention, cognitive control, and emotion regulation (Jung et al., 2014; Vives & FeldmanHall, 2018; Wu et al., 2021). It would be interesting to explore whether ambiguity preferences in different domains are connected to the same or different cognitive functions which can deepen our understanding of the commonalities and distinctions between various ambiguity preferences.

Apart from checking individual differences in ambiguity preferences, we also compared different attribute-wise and integrated-value models in the current study. The debate surrounding these two types of models in multi-attribute choice persists (Busemeyer et al., 2019; Padoa-Schioppa, 2011; Perkins & Rich, 2021) and our results align with several recent studies reporting that attribute-wise models provided better fits than hyperbolic models in intertemporal choice tasks (Amasino et al., 2019; Cheng & González-Vallejo, 2016; He et al., 2023; Scholten & Read, 2010; Scholten et al., 2014). Studies supporting the hyperbolic model, such as Wulff and van den Bos (2018), are in the minority and often limited to relatively small sample sizes. For the risky choices, in contrast, we observed better performance by the integrated-value model over the attribute-wise models. Together, this suggests that participants may employ distinct decision-making strategies in intertemporal versus risky choices.

We observed that in both the best-fitting attribute-wise model for intertemporal choices and the expected utility + additive model for risky choices, ambiguity worked as a distinct, separate attribute without interacting with the reward magnitude. In contrast, the hyperbolic time-perception model and the G-S model posit that ambiguity does interact with reward magnitude. In a prior study with a broader range of reward and ambiguity levels, Ikink et al. (2023) indeed observed such an interaction effect in their choice data, implying that the task design might influence which effects will be identified, either via inducing different decision-strategies in participants, or due to task constraints (e.g., limited power to detect specific effects). Thus, in tasks or designs with more variability in reward and ambiguity, it is possible that models which allow for an interaction between reward and ambiguity might perform better.

It is interesting that among different integrated-value models in the probability domain, we observed that the model which presumes an *additive* influence of ambiguity demonstrated performance comparable to the well-established G-S model. These observations prompt a reconsideration of the prevailing assumptions and suggest the potential existence of models superior to the G-S model for explaining ambiguity preferences in the probability domain.

In conclusion, the current study identified ambiguity aversion within both the time and the probability domain and revealed an association between time-ambiguity aversion and probability-ambiguity aversion, suggesting that ambiguity preferences in different domains might not be completely independent from each other. Furthermore, we observed that participants used distinctive decision strategies in the two task domains—namely, mainly attribute-wise strategies in intertemporal choice and mainly integrated-value strategies in risky choice.

## Supplementary Information

Below is the link to the electronic supplementary material.

Supplementary file1 (PDF 586 KB)

## Data Availability

Raw data are available: https://osf.io/bwnxh/

## Footnotes

1 As the number of models inputted into the PXP calculation increases, the likelihood of encountering a Type I error also rises.

## Code availability

Stan code is available: https://osf.io/bwnxh/
