# Supplementary material for: Ambiguity preferences in intertemporal and risky choice: A large-scale study using drift-diffusion modelling
Source: Psychon Bull Rev. 2025 Jun 25;32(6):2939–56. doi: 10.3758/s13423-025-02709-2 (PMC12627198; doi:10.3758/s13423-025-02709-2)
Supplement: Supplementary file 1 — Supplementary file1 (PDF 586 KB) [file 13423_2025_2709_MOESM1_ESM.pdf]

Supplement

Supplement A: GLMM result with the reduced sample size.

In the tables below, we present the values of the regression coefficients and their 95% HDI obtained from the mixed-effects model analysis with the sample size used for the diffusion model analysis. Notably, while the precise values of these coefficients may differ from the results obtained from the larger participant sample in Supplement B, the conclusions derived from these analyses remain consistent with the conclusions based on the larger sample.

Table S1

Results of the Bayesian linear mixed-effects model analysis with choices in the exact intertemporal choice trials as dependent variable (n = 669).

Regressor	β	95% HDI	ROPE range
Reward	6.03	[5.78, 6.33]	[-0.06, 0.06]
Dealy	-1.95	[-2.06, -1.83]	[-0.06, 0.06]
Reward x Delay	-0.36	[-0.43, -0.29]	[-0.06, 0.06]

Table S2

Results of the Bayesian linear mixed-effects model analysis with choices in the ambiguous intertemporal choice trials as dependent variable (n = 669).

Regressor	β	95% HDI	ROPE range
Reward	4.64	[4.41, 4.85]	[-0.06, 0.06]

Ambiguity	-0.75	[-0.822, -0.67]	[-0.06, 0.06]
Reward x Ambiguity	-0.40	[-0.47, -0.32]	[-0.06, 0.06]

Table S3

Results of the Bayesian linear mixed-effects model analysis with choices in the exact risky choice trials as dependent variable (*n* = 669).

Regressor	β	95% HDI	ROPE range
Reward	3.81	[3.66, 3.95]	[-0.06, 0.06]
Probability	-4.58	[-4.73, -4.46]	[-0.06, 0.06]
Reward x Probability	-2.22	[-2.30, -2.12]	[-0.06, 0.06]

Table S4

Results of the Bayesian linear mixed-effects model analysis with choices in the ambiguous risky choice trials as dependent variable (*n* = 669).

Regressor	β	95% HDI	ROPE range
Reward	3.23	[3.10, 3.40]	[-0.06, 0.06]
Ambiguity	-0.69	[-0.78, -0.61]	[-0.06, 0.06]
Reward x Ambiguity	3.23	[3.10, 3.40]	[-0.06, 0.06]

Table S5

Results of Bayesian linear mixed-effects model analysis with RTs in the exact intertemporal choice trials as dependent variable (*n* = 669).

Regressor	β	95% HDI	ROPE range
Reward	-0.03	[-0.04, -0.03]	[-3.34, 3.34]
Dealy	0.01	[0.01, 0.01]	[-3.34, 3.34]
Reward x Delay	0.01	[0.00, 0.01]	[-3.34, 3.34]

Table S6.

Results of the Bayesian linear mixed-effects model analysis with RTs in the ambiguous intertemporal choice trials as dependent variable (*n* = 669).

Regressor	β	95% HDI	ROPE range
Reward	0.04	[0.04, 0.05]	[-3.69, 3.69]
Ambiguity	-0.02	[-0.03, -0.02]	[-3.69, 3.69]
Reward x Ambiguity	0.02	[0.01, 0.02]	[-3.69, 3.69]

Table S7

Results of the Bayesian linear mixed-effects model analysis with RTs in the exact risky choice trials as dependent variable (*n* = 669).

Regressor	β	95% HDI	ROPE range
Reward	-0.01	[-0.01, 0.00]	[-2.43, 2.43]
Probability	0.05	[0.04, 0.05]	[-2.43, 2.43]
Reward x Probability	0.04	[0.04, 0.04]	[-2.43, 2.43]

Table S8

Results of the Bayesian linear mixed-effects model analysis with RTs in the ambiguous risky

choice trials as dependent variable (*n* = 669).

Regressor	β	95% HDI	ROPE range
Reward	0.03	[0.03, 0.04]	[-3.91, 3.91]
Ambiguity	-0.02	[-0.02, -0.01]	[-3.91, 3.91]
Reward x Ambiguity	0.00	[-0.01, 0.00]	[-3.91, 3.91]

Supplement B: GLMM analysis results.

In Figure 2 of the main text, we presented the posterior distribution of coefficients derived from the mixed-effects model analysis using the full sample (*N* = 1091). Detailed values of these coefficients, along with their 95% HDI ranges, are provided in the following tables.

Table S9

Results of the Bayesian linear mixed-effects model analysis with choices in the exact intertemporal choice trials as dependent variable.

Regressor	β	95% HDI	ROPE range
Reward	5.81	[5.58, 5.99]	[-0.06, 0.06]
Delay	-1.97	[-2.05, -1.87]	[-0.06, 0.06]
Reward x Delay	-0.38	[-0.45, -0.32]	[-0.06, 0.06]

Table S10

Results of the Bayesian linear mixed-effects model analysis with choices in the ambiguous intertemporal choice trials as dependent variable.

Regressor	β	95% HDI	ROPE range
Reward	4.50	[4.28, 4.68]	[-0.06, 0.06]
Ambiguity	-0.73	[-0.80, -0.67]	[-0.06, 0.06]
Reward x Ambiguity	-0.42	[0.49, -0.35]	[-0.06, 0.06]

Table S11

Results of the Bayesian linear mixed-effects model analysis with choices in the exact risky

choice trials as dependent variable.

Regressor	β	95% HDI	ROPE range
Reward	3.75	[3.63, 3.88]	[-0.06, 0.06]
Probability	-4.52	[-4.62, -4.40]	[-0.06, 0.06]
Reward x Probability	-2.17	[-2.23, -2.10]	[-0.06, 0.06]

Table S12

Results of the Bayesian linear mixed-effects model analysis with choices in the ambiguous risky choice trials as dependent variable.

Regressor	β	95% HDI	ROPE range
Reward	-0.75	[-0.80, -0.67]	[-0.06, 0.06]
Ambiguity	3.25	[3.12, 3.38]	[-0.06, 0.06]
Reward x Ambiguity	-0.46	[-0.52, -0.40]	[-0.06, 0.06]

Table S13

Results of the Bayesian linear mixed-effects model analysis with RTs in the exact intertemporal choice trials as dependent variable.

Regressor	β	95% HDI	ROPE range
Reward	-0.03	[-0.01, 0.01]	[-3.33, 3.33]
Delay	0.01	[0.01, 0.02]	[-3.33, 3.33]
Reward x Delay	0.01	[0.00, 0.01]	[-3.33, 3.33]

Table S14

Results of the Bayesian linear mixed-effects model analysis with RTs in the ambiguous intertemporal choice trials as dependent variable.

Regressor	β	95% HDI	ROPE range
Reward	0.04	[0.035, 0.05]	[-4.55, 4.55]
Ambiguity	-0.03	[-0.03, -0.02]	[-4.55, 4.55]
Reward x Ambiguity	0.02	[0.02, 0.03]	[-4.55, 4.55]

Table S15

Results of the Bayesian linear mixed-effects model analysis with RTs in the exact risky choice trials as dependent variable.

Regressor	β	95% HDI	ROPE range
Reward	0.00	[-0.01, 0.001]	[-3.05, 3.05]
Probability	0.04	[0.04, 0.05]	[-3.05, 3.05]
Reward x Probability	0.04	[0.04, 0.04]	[-3.05, 3.05]

Table S16

Results of the Bayesian linear mixed-effects model analysis with RTs in the ambiguous risky choice trials as dependent variable.

Regressor	β	95% HDI	ROPE range
Reward	0.04	[0.03, 0.05]	[-3.34, 3.34]
Ambiguity	-0.02	[-0.02, -0.01]	[-3.34, 3.34]
Reward x Ambiguity	0.03	[0.03, 0.05]	[-3.34, 3.34]

Supplement C: Details of the ROPE ranges.

Traditional null hypothesis testing is susceptible to the issue that, as sample size increases, statistically significant results are always obtained. Yet, the effect size associated with these results may be small, rendering the findings practically insignificant. In contrast, Region of Practical Equivalence (ROPE) analysis enables researchers to discern effects that are practically meaningful. The results of any ROPE analysis are determined by two factors: the size of the ROPE range and the size of the Highest Density Interval (HDI). In accordance with Kruschke (2018), we defined the ROPE as half of a small effect size (using Cohen (2013) cutoffs for a small effect size). In correlation contexts, this value equates to ± 0.05. Conversely, in Gaussian distribution linear regression scenarios, this translates to a range of ± 0.1 standard deviations of the dependent variable.

Kruschke (2018) emphasizes the need to adjust this ROPE range for GLMMs based on the link function. The necessity for a correction in ROPE analysis arises from a fundamental aspect of the generalized linear model (GLM) structure. Consider the GLM equation $y = f(\beta_1 x + \beta_0)$, where $f(x)$ is a link function. In logistic regression for choice modelling, the link function is the inverse of the logit function, whereas in Wald distribution regression for RT modelling, the link function is the exponential function. Here, we are interested in determining the significance of the coefficient β_1. While simply assessing whether the posterior distribution of β_1 includes zero has nothing to do with the link function, ROPE analysis requires more due to its unit-sensitive nature. The coefficient β_1 possesses a unit that must be compatible with the unit of the dependent variable y. To address this, we

transform the posterior distribution of β_1. The first derivative at the intercept is employed for this transformation because it represents the slope of the link function, thus indicating the rate at which the y variable changes. For models based on the Wald distribution, the first derivative of the link function at the intercept is calculated and then multiplied by the raw ROPE range. In logistic regression, following Gelman and Hill's (2006) recommendation, we use the derivative of the inverse of the logit function at 0.5 and apply this to the raw ROPE range.

Furthermore, to get a more robust result, we adopted Makowski et al.'s (2019) suggestion to use the full parameter posterior instead of the 95% HDI for assessing significance. Thus, our criterion for significance entailed having the full parameter posterior completely outside of the ROPE range. However, in the main text we also report 95% HDIs, as this is commonly done.

Supplement D: Parameter recovery results

Parameter recovery is a crucial step in utilizing computational modeling as it evaluates the extent to which we can accurately obtain our simulation parameters through model-fitting (Wilson & Collins, 2019). To assess how well the parameters were recovered, we simulated data using the best-fitting model and each participant's parameter values. For the intertemporal choices, the best-fitting model, determined using PSIS-Loo as the model comparison index, was the attribute-wise model with added ambiguity and an interaction between reward and delay times. For the risky choices, the expected utility + additive model

and the G-S model had nearly identical PSIS-Loo values, we therefore performed the parameter recovery for both models.

Figure S3 shows the parameter recovery of the three best-fitted models. Within the attribute-wise model applied to intertemporal choice tasks, parameters such as the weight for reward (w_r), weight for delay time (w_t), and non-decision time (t_0) exhibit good recovery, with their correlations surpassing 0.9. The boundary separation parameter (A) shows a correlation exceeding 0.85, while both the starting point parameter (z) and the weight parameter for ambiguity (w_{amb}) have recovery correlations above 0.7. However, the weight parameter for the interaction between reward and delay time (w_{inter}) shows relatively poor performance, with a correlation of only 0.56.

In models for risky choice tasks, parameters such as boundary separation, non-decision time, and the starting point parameter show recovery like the attribute-wise model in intertemporal choice tasks. Parameters related to utility computation, including the risk aversion parameter (α), ambiguity aversion parameter (β), and drift rate scaling parameter (η), also exhibit strong performance in both models. However, the G-S model slightly outperforms the EU-additive model in these aspects.

A.

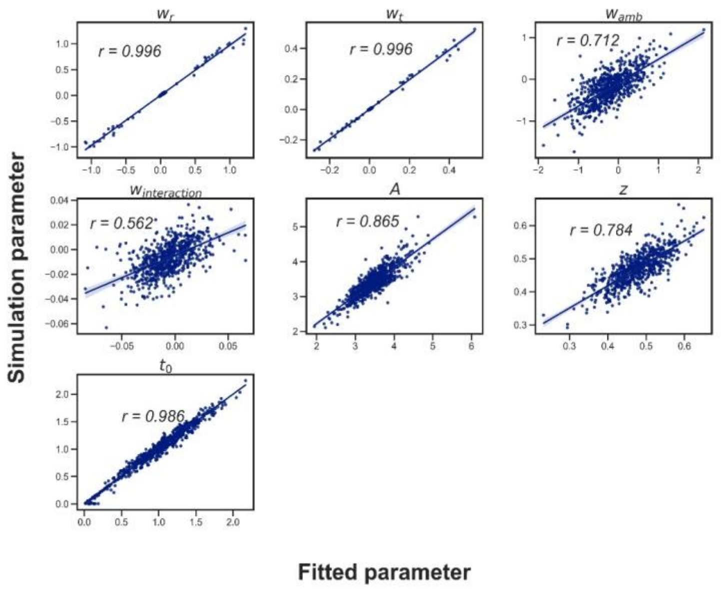

B.

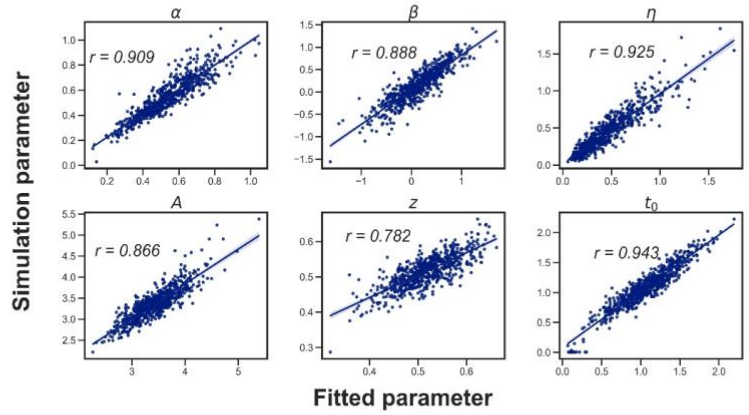

C.

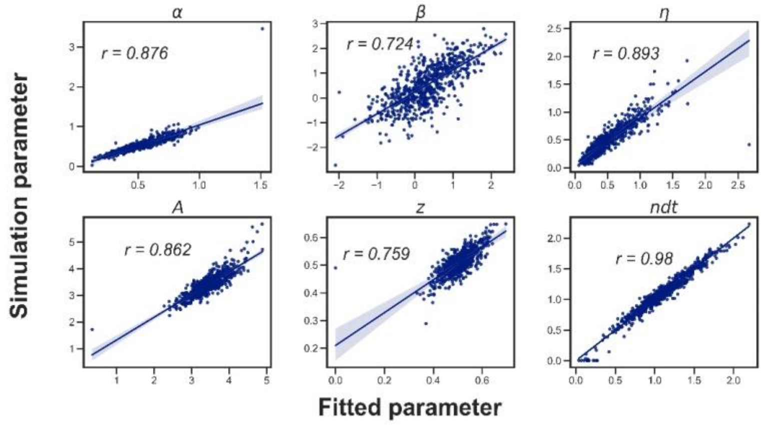

Fig S1. Parameter recovery result**.** The x axis shows the parameter value used for the simulation; the y axis shows the fitted parameter. For the attribute-wise model in intertemporal choice (panel A), for the Gilboa-Schmeidler model in risky choice (panel B) and for the EU + additive model in risky choice (panel C).

Supplement E: Details of the log-model evidence results.

The log-model evidence (LME), also known as marginal likelihood, serves as the foundation for Bayesian model comparisons and Bayesian model averaging (Kass & Raftery, 1995; Wasserman, 2000). The commonly used comparison metric Bayesian Information Criterion (BIC) serves as the approximation of LME (Bishop, 2006; Kass & Raftery, 1995).

LME quantifies the probability of observing the data given a specific model, considering all possible parameter values for that model (Kass & Raftery, 1995). In essence, LME evaluates a model's ability to explain the data by averaging over all possible parameter values, weighted by their prior probabilities.

LME has the capacity to identify the "true model," given it is part of the candidate models, whereas information criteria that approximate cross-validation (eg., AIC, WAIC) aim to select the model whose predictions have the lowest Kullback-Leibler (K-L) divergence from the actual data (Burnham & Anderson, 2004; Gronau & Wagenmakers, 2019; Myung & Pitt, 2018). Also, LME is more sensitive to the prior distribution and exhibits a lower Type I error (Dziak et al., 2020). While cross-validation and log-model evidence differ in various ways, recent studies have demonstrated that log-model evidence is equivalent to cross-validation under certain conditions (Fong & Holmes, 2020; Llorente et al., 2023).

Computing the LME requires integrating the product of the likelihood and prior across the entire parameter space. This process can become computationally challenging, as the integration cannot be solved analytically. Advanced sampling algorithms, such as bridge

sampling, thermodynamic integration, and sequential Monte-Carlo sampling are often employed to compute LME (Annis et al., 2019; Arulampalam et al., 2002; Gronau et al., 2017). Although these methods provide accurate LME estimates, they also demand significant computational resources. In order to reduce the computational burden, we therefore adopted a Kernel density estimation (KDE) approach to compute LME, which offers a less accurate estimate but requires considerably less computation time and effort (Llorente et al., 2021).

We first used the non-parametric classification algorithm k-means to categorize the parameter posterior distribution into three clusters (Thie criterion is based on the previous literature). Next, we applied multivariate KDE to compute the probability of the maximum a posteriori (MAP) estimation $p(\theta_{MAP}|D)$. The bandwidth of the KDE kernel was determined through leave-one-out cross-validation. Finally, LME was estimated using the Bayesian function: $LME = \log \frac{\pi(D|\theta_{MAP}) \times p(\theta_{MAP})}{P(\theta_{MAP}|D)}$, where $\pi(D|\theta_{MAP})$ represents the likelihood function and $p(\theta_{MAP})$ denotes the prior distribution.

Tables S17 and S18 present the median LME and PXP of all model candidates for the probability and time domain, respectively (within tables, models are ordered according to their LME, such that the best-fitting model is shown first in the table).

Tabe S17

Model comparison based on LME in the risky choice task.

	LME (Median)	PXP

Expected utility + additive model	-183.93	1
Gilboa-Schmeidler model	-184.06	0
Expected utility model	-184.29	0
Attribute-wise model with ambiguity with interaction term	-189.52	0
Attribute-wise model + interaction between reward and probability	-192.24	0
Attribute-wise model with ambiguity	-204.28	0
Attribute-wise model with ambiguity with interaction between reward and ambiguity	-205.01	0
Attribute-wise model	-206.87	0

Tabe S18

Model comparison based on LME in the intertemporal choice task.

	LME (Median)	PXP
Attribute-wise model + ambiguity	-186.45	1
Attribute-wise model + ambiguity + interaction between reward and delay	-189.45	0
Attribute-wise model	-189.55	0
Generalized hyperbolic + time-perception model	-192.95	0
Generalized hyperbolic model	-193.04	0
Generalized hyperbolic + additive model	-193.46	0

Attribute-wise model + interaction between reward and delay	-194.29	0
Time-perception model	-206.67	0
Additive model	-207.52	0
Hyperbolic model	-214.78	0

Supplement F: Details on the Inclusion Bayes factor and the Pseudo Inclusion Bayes factor.

Given that each model category, such as ambiguity or non-ambiguity models, comprises multiple models, analyses that rely solely on single-model selection may be affected by model uncertainty. Conversely, Bayesian model averaging and the use of the Inclusion Bayes Factor (BFinclusion) proves beneficial when no single best model can be identified based on model-fitting, permitting researchers to evaluate and compare to each other groups of theoretically meaningful models. Such methodologies have found applications in meta analysis, *t*-tests, ANOVA, and linear mixed-effects models (Heck & Bockting, 2021; Maier et al., 2022; van den Bergh et al., 2020; van Doorn et al., 2021). To compute $BF_{inclusion}$, the Posterior Model Probability (PMP) is needed to combine different models:

$$p(M_k|D) = \frac{p(D|M_k) \times p(M_k)}{\sum_k p(D|M_K) \times p(M_k)} \quad (s1)$$

whereM_k is the Model k and $p(D|M_k)$ is the model evidence and $p(M_k)$ is the model prior. We assigned equal probability to each model prior: $p(M_k) = \frac{1}{N}$, where N is the number of models. The Inclusion Bayes factor is the multiplication between the posterior model odd and the inverse of the prior model odd. For instance, the $BF_{inclusion}$ between ambiguity models and non-ambiguity models is computed as:

$$BF_{inclusion} = \frac{\sum_{M_k \in ambiguity\ models} p(M_k|D)}{\sum_{M_i \in non-ambiguity\ models} p(M_i|D)} \times \frac{\sum_{M_i \in non-ambiguity\ models} p(M_i)}{\sum_{M_k \in non-ambiguity\ models} p(M_k)} \quad (s2)$$

When computing $PBF_{inclusion}$, we substituted PMP with an Akaike type of model weight based on PSIS-Loo (Burnham & Anderson, 2004; Gronau & Wagenmakers, 2019; Wagenmakers & Farrell, 2004). Akaike type of model weight can be computed with a softmax function:

$$w_k = \frac{\exp\left(-\frac{1}{2} Looic_k\right)}{\sum_i \exp\left(-\frac{1}{2} Looic_i\right)} \qquad (s3)$$

Subsequently, $BF_{inclusion}$ between ambiguity models and non-ambiguity models is computed using the following formula:

$$PBF_{inclusion} = \frac{\sum_{M_k \in ambiguity\ models} w_k}{\sum_{M_i \in non-ambiguity\ models} w_i} \times \frac{\sum_{M_i \in non-ambiguity\ models} p(M_i)}{\sum_{M_k \in non-ambiguity\ models} p(M_k)} \qquad (s4)$$

Table S15 presents the inclusion Bayes factor of ambiguity model vs non-ambiguity model and integrated-value model vs attribute-wise model. Note that the pseudo inclusion Bayes factor reported in the main text was computed with Looic whereas the inclusion Bayes factor reported here was computed with LME.

Table S19

$BF_{inclusion}$ results.

Task	Comparison	$BF_{inclusion}$(Median)	PXP	Winning model
Intertemporal choice	Ambiguity model vs non-ambiguity model	8.43	1.0	Ambiguity model
Intertemporal choice	Attribute-wise model vs integrated-value model	13.95	1.0	Attribute-wise model
Risky choice	Ambiguity model vs non-ambiguity model	304.72	1.0	Ambiguity model
Risky choice	Attribute-wise model vs integrated-value model	1.41	1.0	Integrated-value model

Note. PXP represents the support towards the winning model.

Supplement G: Best-fitted DDM parameter value.

The subsequent three tables present summary statistics (mean and standard deviation) of the fitted parameters from the best three models for both the delay and probability domains. The similarity in boundary separation parameters (A) across these domains suggests that individuals exhibit comparable speed-accuracy trade-offs in both areas. Figure S4 shows the correlation between ambiguity weight parameter (w_{amb}) in intertemporal choice task attribute-wise model and ambiguity aversion parameter (β_{amb}) in risky choice task EU + additive model, whereas Figure S5 shows the correlation between ambiguity weight parameter (w_{amb}) in intertemporal choice task attribute-wise model and ambiguity aversion parameter (β_{amb}) in risky choice task G-S model. The two correlation values are similar to each other (0.18 and 0.15).

Tabe S20

Best-fitted parameters for the attribute-wise model with ambiguity and with interaction between reward and time in the intertemporal choice task

	Mean	Standard deviation
w_r	0.036	0.194
w_t	0.008	0.058
w_{amb}	-0.195	0.390
$w_{inter_{ITC}}$	-0.007	0.013
A	3.421	0.408
z	0.471	0.049

t_0	0.991	0.411

Tabe S21

Best-fitted parameters for the Gilboa-Schmeidler model in the risky choice task

	Mean	Standard deviation
α	0.546	0.160
β	0.150	0.397
η	0.462	0.278
A	3.362	0.388
z	0.515	0.045
t_0	1.086	0.356

Tabe S22

Best-fitted parameters for the EU + additive model in the risky choice task

	Mean	Standard deviation
α	0.537	0.198
β	0.502	0.850
η	0.491	0.293
A	3.399	0.416
z	0.511	0.045
t_0	1.078	0.356

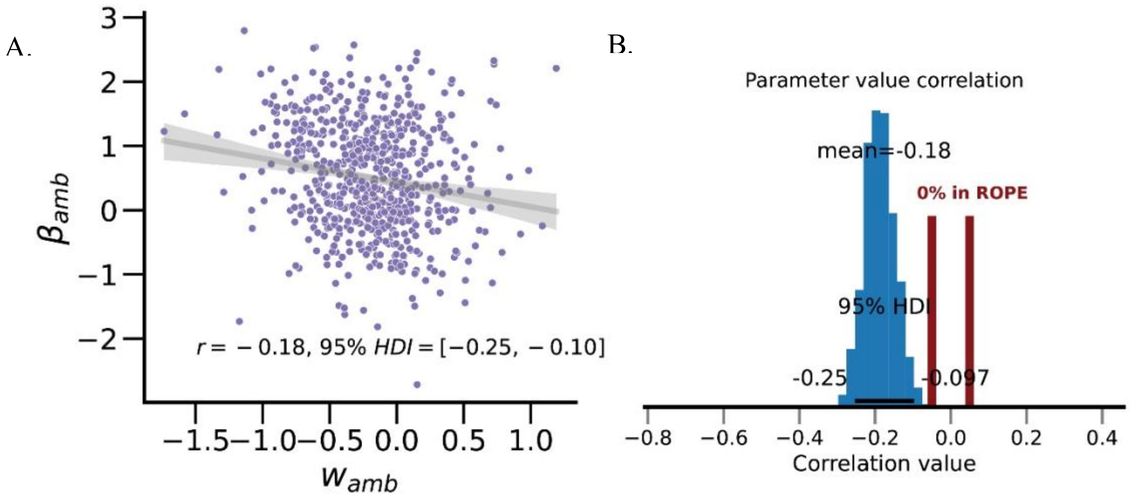

Fig. S2. Examining relationship between ambiguity weight parameter (w_{amb}) in intertemporal choice task attribute-wise model and ambiguity aversion parameter (β_{amb}) in risky choice task EU + additive model. Panel A shows a scatterplot visualizing the correlation of w_{amb} and β_{amb}. Panel B shows the posterior distribution of the estimated correlation coefficient, all samples fall outside of the ROPE range, indicating that the correlation is significant.

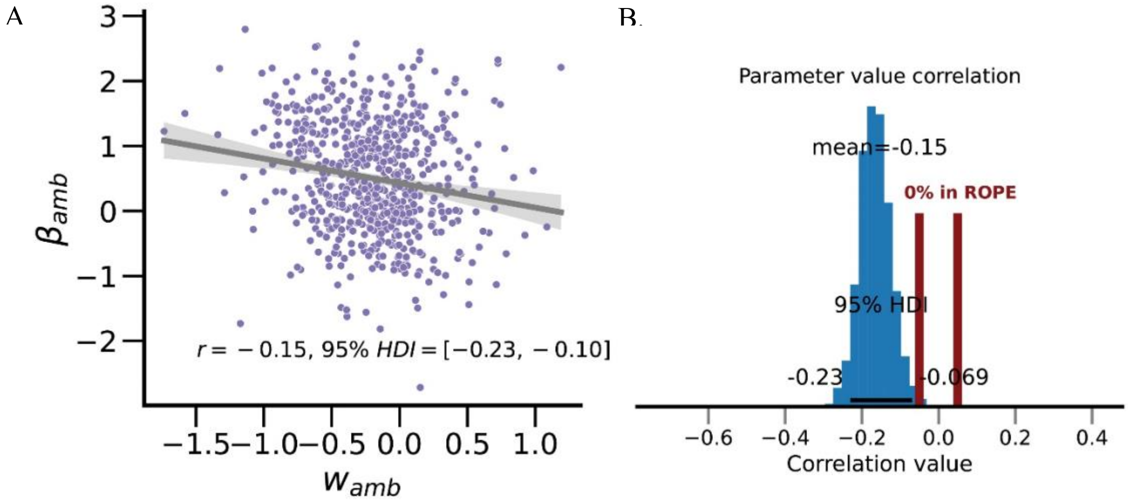

Fig. S3. Examining relationship between ambiguity weight parameter (w_{amb}) in intertemporal choice task attribute-wise model and ambiguity aversion parameter (β_{amb}) in risky choice task GS model. Panel A shows a scatterplot visualizing the correlation of w_{amb} and β_{amb}. Panel B shows the posterior distribution of the estimated correlation coefficient, all samples fall outside of the ROPE range, indicating that the correlation is significant.
